# Confocal Laser Scanning Microscopy and Image Analysis for Elucidating Crumb and Crust Microstructure of Bran-Enriched South African Fried Dough and Batter

**DOI:** 10.3390/foods9050605

**Published:** 2020-05-09

**Authors:** Oluwatoyin O. Onipe, Daniso Beswa, Afam I. O. Jideani

**Affiliations:** 1 Department of Food Science and Technology, School of Agriculture, University of Venda, Thohoyandou 0950, South Africa; 14004637@mvula.univen.ac.za; 2 Department of Biotechnology & Food Technology, Faculty of Science, University of Johannesburg, Doornfontein 2028, South Africa; beswad@uj.ac.za

**Keywords:** *magwinya*, food microstructure, image analysis, fried dough, confocal microscopy

## Abstract

A double staining protocol for image acquisition using confocal microscopy (CLSM) coupled with image analysis was employed to elucidate the crust and cross-sectional properties of fried dough. Penetrated oil by image analysis (POia), porosity and pore features were quantified from the cross-section micrographs. Crust surface roughness was measured using fractal metrics and fat content was determined by solvent extraction using the American Association of Cereal Chemists method. Crumb porosity ranged between 54.94%–81.84% and reduced (*p* < 0.05) with bran addition. Crumb pore sizes ranged from 0–475 µm with <1 circularity, indicating elliptical shape. POia values were notably higher (*p* < 0.05) than PO by Soxhlet extraction (POsox), except for wheat bran (WB) fried dough where the values of POia and POsox were closely ranked. The linear effect of initial moisture content and bran concentration showed a significant impact on the image properties. The mean fractal dimension (FD) decreased as initial moisture increased. The addition of WB caused a significant reduction in the FD of fried dough, while the opposite effect was noted for its oat bran counterpart. Due to non-collinearity of image properties (FD, POia and porosity), data were fitted to cubic polynomial regression with R^2^ values > 0.70. CLSM and image analysis were effective in measuring oil absorption and interpreting crumb properties of fried dough. The protocol used in this study can be applied to other thick deep-fried foods for qualitative observation and quantitative measurement of a specific physical or chemical property.

## 1. Introduction

The transformation of dough and batter into *magwinya* encompasses vital microstructural changes highly dependent on processing conditions such as mixing, and dough development, fermentation (bubble formation as a result of CO_2_ release in the dough/batter), moulding of dough and/or scooping of batter and thermal treatment (frying). The features of breadcrumbs, such as cell wall thickness, cell size, void fraction, porosity and shape have been quantified using image analysis, as reviewed by Pérez-Nieto et al. [1]. The application of confocal laser scanning microscopy (CLSM) for qualitative analysis of food microstructures offers a visualisation of changes to food products, a characterisation of complex food systems, and a distinction between food components [2]. CLSM use in the study of fried foods offers a non-invasive approach to qualitative and measurable evaluation of oil uptake in fried chicken nuggets [3], gluten-based and potato-based food matrices [4]. In addition to oil uptake, quality changes to the texture of the foods, such as crust formation, pore size distribution and porosity measurement, have all been estimated using image analysis [5]. 

The CLSM choice in visualisation of changes in fried foods is due to its ability to produce images with clear contrast, differentiating one food component (fat, protein or carbohydrates) from the other and from the empty pores. This is achieved using dyes specific to a food component and simultaneously viewing these components. Staining post-frying has been used, but this could yield unreliable results due to the timing of staining which does not reveal oil uptake relative to frying unit operation. Moreover, the formation of artefacts occurs, leading to errors in the data analysis from the resulting image. Instead, staining the food and oil prior to frying is a better step in sample preparation which excludes post-processing staining [6]. The choice of fluorophore depends on its affinity to the food component of interest, emission spectra, and behaviour in the food matrix [4,7]. 

Quantitative analysis of food products observed microscopically has become a vital tool to evaluate the quality of products. In the same way food is processed through the application of different unit operations, significant information can be extracted from an image by subjecting it to processing through mathematical operations on the raw image [8,9,10,11]. Raw and processed foods have irregular and complex geometries and, as such, can be quantified using fractal metrics—a useful tool to measure the surface topography of various objects, including food [12,13]. In fried food research, fractal analysis has been used to measure surface roughness [5] and correlated to oil absorption [12,13,14]. The surface roughness of foods is quite sensitive because what looks smooth to the human eye may be jagged when examined microscopically. Fractal dimension (FD) measures how much space is filled in an image using thresholding and edge detection tools [9].

*Magwinya* is a deep-fried dough/batter common to sub-Saharan Africa and has recently been enriched with wheat bran [15], oat bran and psyllium husk fibre [16] for oil uptake reduction, and a descriptive sensory profile [17]. However, the application of CLSM to study the microstructure and surface topography of fried dough (*magwinya*) has not been carried out for quantitative evaluation. Therefore, the aim of this paper was to extract meaningful empirical information from the crust and cross-section confocal micrographs of *magwinya* in order to: (1) observe and enumerate the penetrated oil, porosity and pore properties of the crumb; (2) determine the surface roughness of crust as affected by bran levels in *magwinya* formulation, using fractal metrics; and (3) evaluate the effect of surface roughness on surface and absorbed oil contents and assess the relationship between surface roughness and crust texture. 

## 2. Materials and Methods

### 2.1. Materials

Oat bran (Tiger Brands), wheat bran (Snowflake), sunflower oil (Spar) and wheat flour (Sasko) were all sourced from South Africa. Nile Red-72485, (Sigma Aldrich, MO, USA), fluorescein-5-isothiocyanate (Fluka, Switzerland) and petroleum ether (40–60 °C) were of analytical grade. Wheat and oat bran (OB) were pulverised with an ultra-centrifugal mill (Retsch ZM 200, Haan Germany) fitted with a 200 µm sieve. Milled bran was substituted and blended in wheat flour at 5%, 8%, 10%, 15% and 20%. 

### 2.2. Fried Dough (Magwinya) Production and Frying Process

The depth of oil in the fried products was observed with confocal laser scanning microscopy (CLSM), according to the non-invasive double staining method of Moreno and Bouchon [4]. For proper differentiation between oil and other components of the samples, two types of dyes were used—fluorescein-5-isothiocyanate (FITC) (for batter and dough staining) and Nile red (for oil staining). Fluorescein-5-isothiocyanate (FITC) was chosen for dough staining because it stains starch and gluten well. It works on the principles of hydrophobicity—that is, its ability to gather in hydrophobic regions, such as gluten rich portion of the dough [4]. Nile red was chosen for oil staining for its fat-soluble qualities, thermo-resistance, and emission spectra which are clearly distinct from FITC [4]. The staining procedure was done in two phases.

**Phase 1:** Dry ingredients (flour 100 g, yeast 1 g, sugar 15 g, salt 1 g,) were weighed and mixed thoroughly. About 0.01% *w*/*w* FITC was dissolved in distilled water at 20 °C for 1 h by agitation using a magnetic stirrer. The FITC-stained water (65 mL and 100 mL) was added to the dry ingredients for dough and batter preparation, respectively, for identification of the solid matrix during CLSM observation, without further staining post-frying [4]. 

**Phase 2:** The second staining was carried out by dissolving Nile Red at 0.05 g/L in the frying oil in order to identify oil distribution in the *magwinya* during CLSM observation. The FITC-stained dough and/ or batter was deep-fried in the Nile red-stained oil at 180 °C for 5 min. The fried *magwinya* was cooled to ambient temperature in the desiccator until imaging. 

### 2.3. Microstructural Observation

The cross-section and crust of samples were observed with a LSM780 confocal microscope (Zeiss, Oberkochen, Germany) equipped with an Argon multiline laser at 200 µm depth. Four thin cross-section slices (0.5 × 0.5 × 0.5 cm) were cut from each fried product and directly viewed under the lenses of the microscope, with two channels of observation of the confocal microscope in fluorescent mode. The FITC-stained solid matrix (dough and batter) was observed in channel one, while the Nile-Red-stained oil showing fat distribution was viewed in channel two after exciting at 488 nm and 543 nm, respectively with the Argon laser. Images were acquired by setting the objective lens at 10× magnification with numerical aperture of 0.3 over regions of 512 × 512 pixels. The two-dimensional (2D) stack of images was collected to produce 8-bit 2D images in Carl Zeiss image (CZI) format at 2.768 µm/pixels in x and y directions. Images were collected to a computer using the ZEN 2.3 SP1 software (Carl Zeiss, Oberkochen, Germany 2012), which was used for image acquisition, simple image processing (using the maximum intensity projection for image enhancement, and best fit function for optimal contrast between the two channels) and documentation. ZEN software also offers the option of saving the images in choice format. Images saved both in CZI and Tagged image file (TIF) format on the ZEN software were then exported to ImageJ version 1.52q (National Institute of Health, Bethesda, MD, USA) for further analysis.

### 2.4. Image Segmentation and Analysis

Image segmentation was done using ImageJ software [18,19]. The stack composite was opened in software using the split channel function in the hyper stack plugin. The red channel was analysed for fat distribution, while the green channel was analysed for the solid matrix. Automatic thresholding was done in ImageJ using the Otsu algorithm, followed by two steps of erosion and dilation to remove noise [3,20]. Demarcation between the pore and rest of the image was done in one of two ways: (1) the TIF version of the composite image was opened in ImageJ software, and the pore area (in black) were manually selected by holding down the SHIFT key and the lines were traced around the boundaries of the pores; and/or (2) using the wand tool (at a connectedness of four and a tolerance level between one and five) to automatically select the pore areas. The wand tool creates a selection by tracing objects of uniform colour or similar pixels. The selected areas were then filled with an alternate colour (blue) prior to thresholding with the Triangle algorithm in ImageJ software. Triangle algorithm was used because it gave better pore segmentation from the solid matrix, with minimal noise. The segmented images were analysed for pore circularity (Equation (1)), particle count (ΣP), total area (ΣA), average size (ØA), solidity and perimeter (P) using the analyze particle function. Porosity and penetrated oil by image analysis (POia) were calculated using Equations (2) and (3) [3,4].

(1) $$\mathrm{Circularity}\;(C)=4\pi \;\frac{\mathrm{Area}}{{\mathrm{Perimeter}}^{2}}$$

(2) $$\mathrm{Porosity}\;(\%)=\frac{\mathrm{Area}\;\mathrm{of}\;\mathrm{empty}\;\mathrm{pores}+\mathrm{Areas}\;\mathrm{of}\;\mathrm{pores}\;\mathrm{filled}\;\mathrm{with}\;\mathrm{oil}}{\mathrm{Total}\;\mathrm{image}\;\mathrm{area}}\;\times \;100$$

(3) $$\mathrm{POia}\;(\%)=\frac{\mathrm{Area}\;\mathrm{of}\;\mathrm{pores}\;\mathrm{filled}\;\mathrm{with}\;\mathrm{oil}}{\mathrm{Total}\;\mathrm{image}\;\mathrm{area}}\;\times \;100$$

### 2.5. Quantification of Surface Roughness of Magwinya Using Fractal Analysis

The surface roughness of the crust of *magwinya* was quantified with the box-counting method through the fractal box count algorithm of Image software. The crust micrographs were opened in the software and were binarized. Erosion and dilation operations were applied to remove noise from the images. Thereafter, the edges of the images were automatically found with a Sobel edge detector. This operation distinguished the edge from the background. The fractal dimension (FD) of the edged image was calculated by applying the ‘fractal box-count plugin’ which counts the number of boxes of increasing sizes (2, 3, 4, 6, 8, 12, 16, 32 and 64) needed to cover the boundary of binary object per pixel and applies the method [21]. A graph of the logarithmic values of the fractal box size vs. the count was plotted and fitted linearly. The slope of the graph taken as fractal dimension (Equation (4)). The value of the slope is explained as the surface roughness index for the surface.

(4) $$D=-\frac{log\left(N\right)}{log\left(r\right)}$$

where *N* is the number of boxes, and *r* is the size (length) of the boxes and D (slope of the graph) is the fractal dimension. As proposed by Rahimi and Ngadi [13], because the FD was quantified from a 2D space, the addition of one extra dimension is necessary to fully capture the three-dimensional features of the images of the batter surfaces. Therefore, FD was recalculated as per Equation (5).

(5) FD=1+D

### 2.6. Determination of Oil Content by Soxhlet Extraction

Fried products were dried to a constant mass and pulverised in a grinder. About 5 g of ground sample was transferred into 25 × 80 × 1.5 mm cellulose extraction thimbles (Whatman Intl. Ltd., Maid stone, UK). Extraction was done in an automated Soxhlet machine using petroleum ether (40–60 °C) for 4 h based on the AACC method 30–25.01 [22]. 

### 2.7. Statistical Analysis

All images were acquired in a replicate of four per frying time. Analysis of variance was carried out to determine the effect of bran concentration on the porosity, POia and fractal dimension. Means were separated using Tukey’s honestly significant difference test at *p* < 0.05 where the effect of the independent variable was significant. A test of significance for the FD of fried batter and dough was carried out using an independent *T*-test, at a 95% confidence level [4]. Multivariate regression analysis was carried out to determine the linear and interaction effect of bran addition, initial moisture content and bran type on dependent variables. The relationships between crust parameters and the FD of the fried products were fitted through a regression model using the curve estimation function of SPSS software (SPSS statistics version 26, IBM Co., Armonk, NY, USA).

## 3. Results and Discussion

### 3.1. Qualitative Analysis of Microstructure

**Figure 1:**
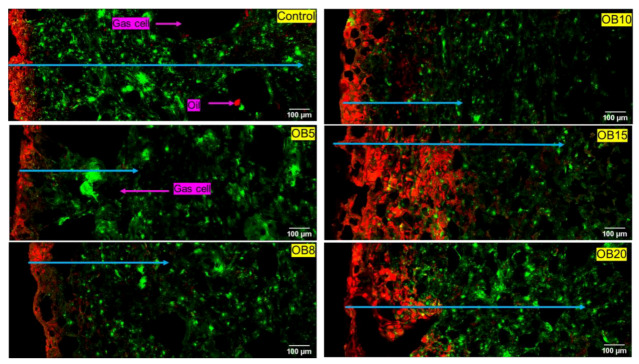
Cross-section micrographs of fried dough enriched with oat bran (OB). Red = oil, green = dough matrix. Blue arrows indicate depth of oil penetration in the solid matrix. OB5–OB20 represent oat bran concentration (g) in product formulation.

During preliminary experiments, the samples were stained with Nile red after frying and this led to the formation of artefacts. Oil and water droplets moved from samples to the microscope slide, thus yielding unreliable results. Therefore, the double staining protocol of dough and oil staining was used because it yielded a satisfactory contrast between the oil (red), empty pore (black) and the solid matrix (green), as shown in Figure 1. The enhancement of the image contrast offers good benefits in terms of the information that can be extracted from such an image [23].

#### 3.1.1. Oat Bran (OB) *Magwinya*

**Figure 2:**
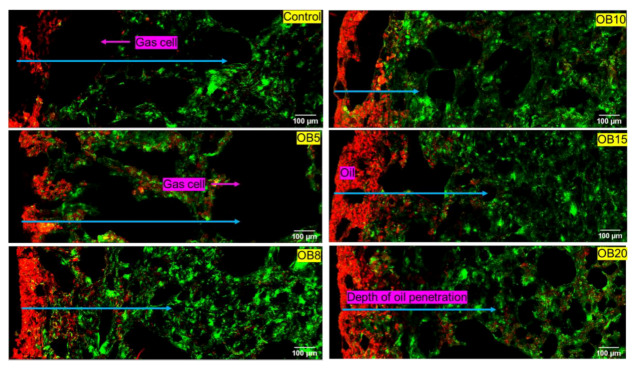
Cross-section micrographs of oat bran fried batter enriched with oat bran (OB). Red = oil, green = batter matrix. Blue arrows indicate depth of oil penetration in the solid matrix. OB5–OB20 represent oat bran concentration (g) in product formulation.

Cross-sectional micrographs of OB fried dough are presented in Figure 1. As can be noticed in all samples, there is intensity of red colour (oil) clusters at the crust section and subsequent penetration at different depths into the solid matrix - indicated by the diminishing intensity of red dye (or blue arrows) from the crust to the crumb in the solid matrix, thus confirming that oil uptake in *magwinya* is a surface-related phenomenon following a crust-to-crumb path flow. Depth of oil penetration shows a similar proximity to the crust at 5 and 8 g and further from the crust at 10–20 g OB. It is worth noting that the sample size used for microscopy may not reflect the whole sample compared to conventional techniques. Pores in fried dough (Figure 1) were small and almost evenly distributed while in fried batter (Figure 2), the pores were mostly large. Large pores are indicative of a weak gluten–starch matrix and thin liquid lamellae caused by reactions between surface active agents of the dough and gluten network, which collapses upon heating, leading to the formation of large coalesced gas cells in the crumb [24]. This consecutively causes large pathways in the dough for oil penetration—hence the increased depth of penetration compared to fried dough samples, which had better stronger structural integrity. These qualitative observations are supported by the Soxhlet extraction results, where fried batter absorbed more oil than fried dough samples.

#### 3.1.2. Wheat Bran (WB) *Magwinya*

**Figure 3:**
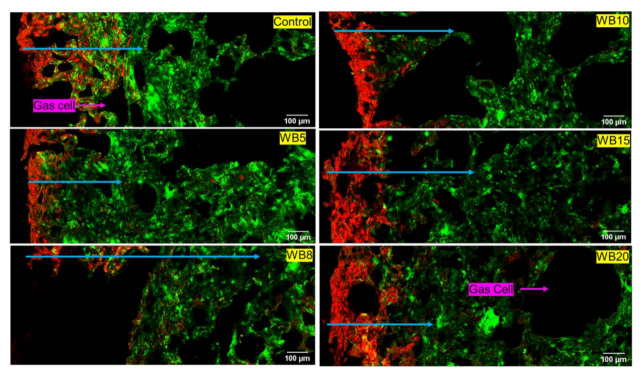
Cross-section micrographs of fried batter enriched with wheat bran (WB). Red = oil, green = batter matrix. Blue arrows indicate depth of oil penetration in the solid matrix. WB5–WB20 represent wheat bran concentration (g) in product formulation.

**Figure 4:**
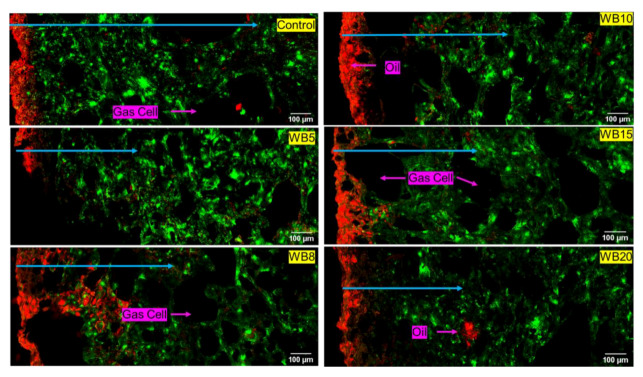
Cross-section micrographs of fried dough enriched with wheat bran (WB). Red = oil, green = dough matrix. Blue arrows indicate depth of oil penetration in the solid matrix. WB5–WB20 represent WB concentration (g) in product formulation.

The depth of oil penetration was more in fried batter (Figure 3) than fried dough (Figure 4). Comparing both cereal brans, the least oil penetration was observed in WB-enriched *magwinya*. This implies that WB reduced oil uptake better than OB. Generally, fried batter had larger geometric features than fried dough. The differential water contents and viscosity of dough and batter (liquid dough) explains the reason for the structural differences among the samples. Like its OB counterpart, large pores were also observed in WB fried batter. Pores of *magwinya* develop and increase as steam is generated in the crumb. Subsequently, crumb pressure and temperature drop during cooling and pores are stabilised as they assume their final shape. In addition to these visual observations, measurable comparisons made using quantitative image analysis are presented in the subsequent section. Quantifiable data (porosity, pore size, penetrated oil) were extracted from the micrographs using Image J software.

As the onset of fermentation, gas cells expand, and their stability hinges on the viscoelastic gluten starch matrix. However, later in the fermentation process, on liquid lamellae is formed from flour surface active components like lipids, polysaccharides and proteins. The liquid lamellae act as a dual protection on each side of the cell wall to prevent the rupturing of the gas cells [24]. In batter products with high moisture content, the gas cells become highly discontinuous, thus leading to the formation of large holes in the final product, as seen in fried batter products in this study. However, bran addition in batter formulation reduced large pores in the *magwinya* crumb at 8–20 g OB (Figure 2) and 15–20 g WB (Figure 3). This is because soluble fibres have been reported to strengthen dough structure. In this case, beta glucans in OB caused a gel-like structure which contributed to improvement of the *magwinya* crumb. On the other hand, insoluble fibre in WB has been reported to lower gas cell stability through interference with gluten reaggregation, thus impacting the dough negatively [25,26]. The large crumb pores in WB fried batter suggests that the negative effect is related to increased surface area of fine WB, which accelerated chemical interactions with gluten. Components like phytates, glutathione and monomers of conjugated ferulic acid binds to the cell wall of the insoluble fibre, thus altering the functionality of gluten network to stabilise the gas cells [26]. 

### 3.2. Penetrated Oil Content by Image Analysis

Quantitative data were extracted from the micrographs as presented in Table 1 for porosity, penetrated oil by Soxhlet extraction (POsox) and image analysis (POia). Fried batter showed significantly higher POia values (*p* < 0.05) than fried dough for WB and OB *magwinya*. 

#### 3.2.1. Oat Bran *Magwinya*

The POia values for OB-enriched fried batter and fried dough were in the ranges 14.52%–18.22% and 5.56%–18.92%, respectively; while POsox values for OB fried batter and dough were in the ranges 7.47%–9.47% and 5.27%–8.20% The POia values for fried batter were significantly lower than the control (19.66%) except for OB15 (18.22%) while the opposite trend was observed for the fried dough. POia was significantly higher (*p* < 0.05) than POsox for all samples except for the control and OB5 fried dough, possibly due to the small amount of bran, thus showing no significance from the control. In fried batter, OB5–10 and OB20 samples were not significantly different (*p* < 0.05) from each other, but were markedly lower than the control and OB15. In OB fried dough, a reverse trend was observed. A similar trend was observed in POsox results (Table 1). POia values at OB8–20 for fried dough samples were significantly higher than the control. During frying, moisture evaporates from the product, creating crevices which serve as pathways for oil influx into the food. This reduction effect of OB observed in *magwinya* may be linked to the water retention capacity of OB fibres and the slight gelling effect of OB β-glucan, which impedes moisture loss and, in turn, reduces oil uptake in the products. Results of POia and POsox for OB fried batter and POsox for fried dough were similar to that of Yadav and Rajan [27], where a significant reduction in the oil content of Indian deep-fried dough (poori) was reported at 11 g OB inclusion. The effect of OB in the products differed based on the initial moisture content which affected moisture loss and, in turn, oil uptake in the products. Either by conventional Soxhlet extraction or image analysis, both methods are valuable for the quantification of the oil uptake of deep-fried products.

#### 3.2.2. Wheat Bran *Magwinya*

The POia of WB fried dough and batter ranged from 4.57% to 9.29% and 13.18% to 16.00%, while POsox values for WB fried dough and batter ranged from 5% to 8% and 7.67% to 10.13%, respectively (Table 1). The results of oil content were comparable in WB fried dough for both image analysis (4.57%–9.29%) and Soxhlet extraction (5%–8%). Moreno and Bouchon [4] reported a similar trend where the oil content of gluten fried matrices was ranked similarly by CLSM and Soxhlet extraction methods. On the other hand, POia was markedly (*p* < 0.05) higher than POsox in WB fried batter. A significant reduction was noted in POia from the control to WB10 and an increase beyond this in the fried batter. This implies that WB reduced oil penetration in fried batter up to 10 g substitution. In fried dough, oil uptake reduction was noticed at WB10–20; and can be linked to a reduction in moisture loss because of the increased bran concentration. Yadav and Rajan [27] reported a significant reduction in poori at 3 g incorporation of coarse WB. Particle size reduction in WB in this study (200 µm) could account for the variation in these results. Moreover, the results of this study were comparable to those of Kim et al. [28], where fat reduction in doughnuts were significant at 10 g WB (60.7 µm) incorporation. The oil uptake reduction effect of WB in *magwinya* can be attributed to the particle size reduction, which lowered its oil holding capacity, which is related to the hydrophilic nature and change in density and surface properties of the bran particles [28]. The insoluble fibre of WB potentially hinders oil uptake. Based on these data, low-fat *magwinya* can be produced from wheat flour- bran composites. While Soxhlet extraction is a conventional, destructive and invasive method, image analysis is a non-destructive technique, where the samples can be imaged whole or in sections. However, the information collected was restricted to the image resolution, which accounted for differences in results. 

The effect of frying time and temperature on oil location in potato chips using CLSM was studied by Pedreschi and Aguilera [29]. Although only a visual observation was made, crucial information regarding the mechanical effect of cutting potato and its implication on oil uptake was reported. As previously stated, oil absorption is predominantly a surface-associated phenomenon. Bouchon et al. [6] demonstrated this phenomenon by observing a fried potato slice under a CLSM, and it was noted that oil absorbed post-frying was in the crust microstructure, as observed in this study. Moreno and Bouchon [4] developed the double staining procedure where the dough matrix and oil were both stained prior to frying. A relationship was established between the microstructure of the products and the oil absorption by comparing the values of fat content from Soxhlet extraction and image analysis. A positive correlation was established between the results. Moreover, porosity, and pore size distribution were estimated from the confocal images and a direct proportional relationship was established between oil absorption and porosity of a gluten-based product, revealing that higher porosity led to higher oil absorption. Fat distribution, pore sizes, surface topography and the porosity of fried chicken nuggets were also estimated using CLSM. However, the samples were stained with Nile A and images were collected in reflective and fluorescent mode for the determination of surface topography and fat distribution in the chicken nuggets. There was a positive correlation between the results from the CLSM image analysis and conventional Soxhlet fat extraction [3]. 

### 3.3. Porosity of Magwinya

**Figure 5:**
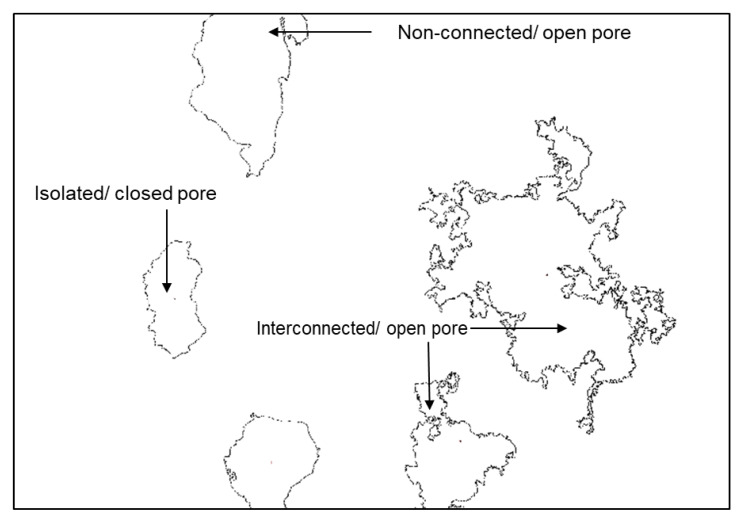
Types of pores identified in fried dough (*magwinya).*

Porosity is a measure of the void fractions in a material [30]. These voids can be closed or open, connected or disconnected (Figure 5). Control fried dough and batter were highly porous at values ≥80%. Bran addition significantly reduced porosity of the products. The porosity of OB and WB fried dough ranged from 54.94% to 69.21% and 52.15% to 73.08%, respectively, and were significantly lower than the control fried dough (80.03%). The porosity of OB and WB fried batter ranged from 65.91% to 80.86% and 53.12% to 76.05%, respectively and were significantly lower (*p* < 0.05) than the control fried batter (81.84%), except for OB8–OB15 (Table 1). The overall effect of bran type showed no significant effect on porosity (*p* > 0.05). These results majorly imply that bran incorporation improved the crumb structure of the products. Large pores associated with high porosity values are characteristic of gas cell collapse during heat treatment (frying). With bran addition, gas cell coalescence reduced, most especially in fried dough products. The higher range of porosity in fried batter may be due to the higher coalescence of gas cells. This reduction effect may be linked to the increased viscosity of the batter and dough imposed by increasing bran concentration, as also observed by Sabanis et al. [31]. Statistically, bran type showed no effect (*p* > 0.05) on porosity, whereas initial moisture content and bran concentration showed a significant reduction effect (*p* < 0.05) on porosity. In addition, the interaction of bran type × bran concentration × initial moisture content was a statistically significant factor of variation in the porosity of the samples (Table 2). Porosity reduced significantly in fried dough compared to fried batter. 

The porosity values in this study fall within the range reported in the literature for similar foods such as bread [31,32] and doughnuts [33]. However, the variations in these values may be attributed to different image acquisition methods and scales used in analysis. The use of X-ray microtomography by Wang et al. [32] showed bread crumb porosity values (79% – 84%) in the same range as values in this study. The use of microscopic image acquisition may account for this similarity. The use of digital camera imaging in the study of Ghaitaranpour et al. [33] may have accounted for the lower porosity profile of deep-fried doughnut crumbs in the range of 54%–66%. Moreover, ingredients in their doughnut formulation, like eggs, gluten, xanthan gum, milk powder, vegetable oil, accounted for differences in the dough rheology, which impacted the aeration and stability of cell sizes in the doughnut crumb. 

The mechanism of pore development in *magwinya* follows water movement from the core to the evaporation zone at the crust followed by its dissipation from the product as vapour. However, remnant vapour left within the pores becomes superheated and expands, causing the distortion of the pore walls [34], hence contributing to the porosity development of *magwinya.* Regression analysis revealed that the linear effect of independent variables (bran type, initial moisture content and bran concentration) showed significant effects on the POia of fried products. The interaction effect of the independent variables showed a significant effect (*p* < 0.05) on POia (Table 2).

Similar to bread, pore development in *magwinya* is influenced by product ingredients and processing unit operations, as follows: yeast as a leavening agent causes bubble formation in the dough. This leads to formation of gluten–starch matrix, characterised by a network of pores that are fully developed during mixing, moulding and fermentation unit operations [23]. The onset of gas cells begins during the mixing of ingredients through aeration or the incorporation of air into the dough matrix. As heat is applied during frying, the porous structure becomes stabilized, which causes modification of the molecular arrangement of the polymers in the cell wall. 

### 3.4. Pore Distribution

Three types of pores which may influence oil penetration have been identified [34] in fried products: (a) interconnected pores—these are accessible from various points and greatly influence the flow of oil due to the continuous paths formed by the interconnection of the pores, (b) non-connected pores, which are inaccessible and do not influence flow of oil through the food matrix, (c) isolated pores, which are accessible from just one direction and have limited influence on oil flow. Non-connected and inter-connected pores are open, while blind pores are closed pores (Figure 5). These pores were identified in our products. 

**Figure 6:**
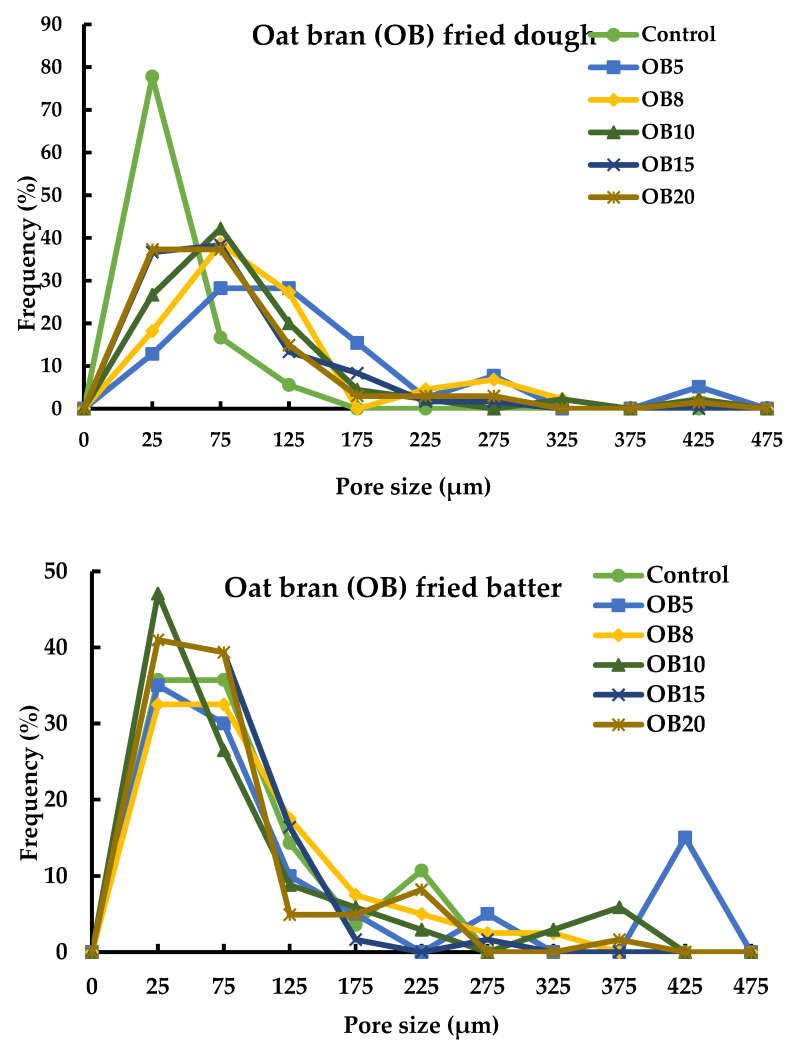

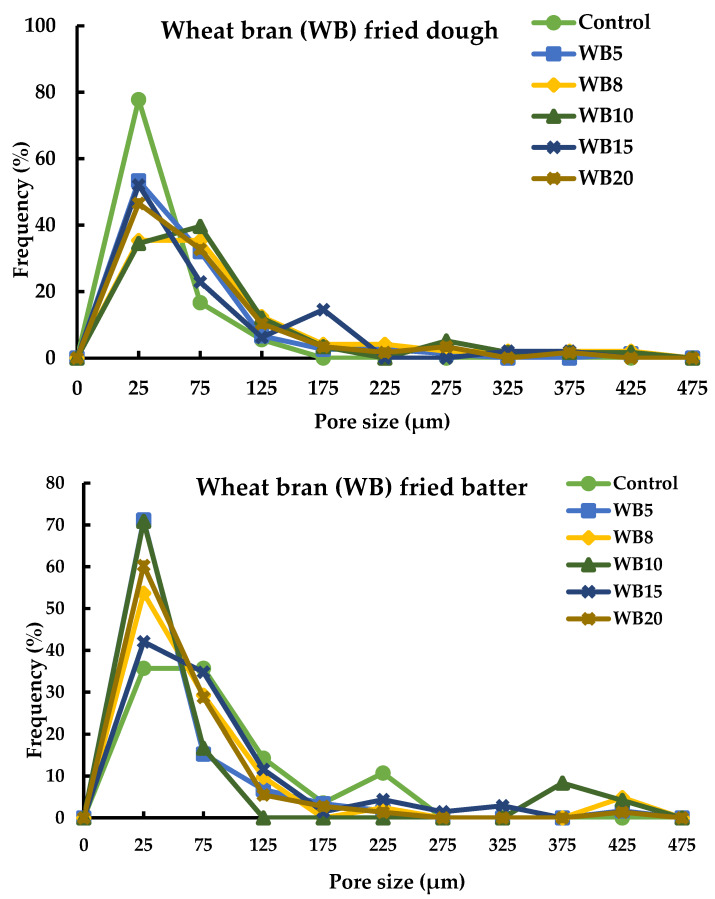
Pore size distribution in cross-section micrographs of fried dough and batter samples. Oat and wheat bran (OB and WB, respectively); values between five and 20 represent the concentration of bran in the sample formulation.

Pore sizes of magwinya obtained from cross-section micrographs were found in a range of 0–475 µm (Figure 6). In fried dough samples, the control pore sizes between 0–25 µm had the highest frequency (80%), while in fried batter samples, control pore sizes of 0–25 µm peaked at 35% and 50% for OB and WB fried batter, respectively. For the rest of the samples at 5%–20% bran addition, pore sizes of 0–175 µm peaked at 52%, 40%, 60% and 47% in WB and OB fried dough, WB and OB fried batter, respectively, then dipped at >125 µm size. Moreno and Bouchon [4] reported a pore size range of 0–85 µm for gluten-based fried matrices. These differences in pore sizes could be accounted for by the following variations: (i) sample weight and size variation—thick products (at least 50 g weight and > 50 mm diameter) in this study and thin products in theirs (4 g weight, 2 mm thickness), (ii) the extent of sample dehydration/moisture loss which contributes to the enlargement and shrinkage of pores. At a final moisture content of 2% in the report of Moreno and Bouchon [4], the associated extreme shrinkage of pores could be explained by maximum moisture loss and total starch gelatinisation, which led to the alteration of gas cell sizes formed during mixing and kneading unit operations.

*Magwinya* consists of a solid medium of interconnected webs of pores, filled with either oil or air. The properties of empty pores of *magwinya* are presented in Table 3. After segmentation of the image using the wand tool in the hue-saturation-brightness colour space, the number of pores in each image was estimated with the analyse particle function. 

Fried batter was characterised by large pores evidenced by the cell size (Table 3). Circularity is a shape descriptor, defined as the ratio of the area of an object to the area of a circle with the same perimeter and it is also known as the compactness of an object/shape [35,36]. It describes how perfect to a circle an object is, because a circle is a compact shape. A value of one describes a perfect circle, while zero describes an elliptical/ irregular shape. A pore with perfect circle with no connectedness to other pores is an isolated pore and will not aid oil flow, whereas irregularity of the pores may aid in oil flow. Solidity is another shape descriptor that measures the density of a particle, which is derived from the ratio of the area to the convex hull area of a particle. A solidity value of one means the particle is a solid object and value less than one represent an object with an irregular boundary [35]. 

#### 3.4.1. Pore Distribution in Fried Dough

The empty pores of *magwinya* were found in a broad range of sizes (0–475 µm), as gas cells in an uninterrupted foam-like structure. The particle counts of the empty pores of fried dough samples are shown in Table 3. Pore particle count increased with an increase in bran concentration (Table 3). Bran incorporation facilitated the even distribution of gas cells in the samples. The particle count in fried dough was higher than fried batter, which implies an even distribution of pores in the former, due to the incorporation of air during kneading. Solidity values of 0.64–0.77 in fried dough samples fall into the category of the star-shaped particles described by Wirth [35], while circularity ranged from 0.13 to 0.39. The total area (11.98–28.97 × 10^4^ µm^2^), average size of the gas cells (727.79–6120.47 µm^2^) and perimeter (150.14–1119.44 µm) are presented in a wide range of sizes in the samples (Table 3). The circularity of fried dough samples (0.10–0.29) were all less than one, which denotes an elliptical shape for *magwinya* pores.

#### 3.4.2. Pore Distribution in Fried Batter

The particle counts of pores increased with bran increase in fried batter samples, especially at 15% and 20% (Table 3). Circularity was highest in the control at 0.42, meaning the pores in the control sample were less elongated compared to the rest of the samples. Similarly, solidity was highest in sample OBB8 at 0.84, meaning the pores had boundaries close to a perfect solid object (circle). Solidity was higher in OB samples at 0.72–0.81 than WB samples (0.66–0.74). Other properties, like total area (17.69–42.03 × 10^4^ µm^2^), average size (2691–21016 µm^2^), and perimeter (310.75–860.90 µm), were found in a wide range of sizes across WB and OB concentrations. Fried batter was characterised by large pores linked to the rupture of gas cells as a result of a weak gluten network in the batter, because stability against coalescence is maintained when the gluten film expands biaxially without rupturing [37]. The extent of the expansion of gas cells at the frying stage determines the final volume and crumb structure of the fried products. Shearing/disruption of the pore network could also occur as a result of mechanical stress induced during the moulding of dough and the cutting of the fried sample for imaging. These pores are an interconnected network which can be empty or filled with oil. 

Unlike breadcrumbs made up of two phases (air and cell wall), *magwinya* crumb is made up of three phases—a fluid (oil), a solid (cell wall material) and a gas (air) phase. Aeration of the magwinya dough/batter matrix may occur through one or both of two ways: (i) physical aeration as a result of air entrapment in the dough during mixing of the batter and kneading of the dough and (ii) biological aeration of the gas cells due to the action of baker’s yeast, *Saccharomyces cerevisiae*, which releases CO_2_ in the dough and causes an almost ten-fold increase in the air incorporated at the mixing stage [38]. Air is entrapped into the porous structure during mixing [39]. The nuclei for gas cells found in the breadcrumb is majorly formed at this stage and is reliant on the mixer type used [23]. The air entrapped during mixing forms the basis of the cell size in the crumb, especially kneading, which promotes an increase in gas volume fraction. An increase in gas cell size as fermentation commences in bread dough has been reported [39,40]. Considering this and the type of mixing in this study—manual mixing (use of spatula) for batter and mechanised mixing (dough mixer) for dough—air incorporation and occlusion differed in both dough types and the final crumb structure of *magwinya.*

The homogeneity of the foam structure during dough development is influenced by gravitational force and pressure differences exerted by CO_2_ during gas cell expansion at various points in the dough mass. Where gravitational force is negligible or insignificant, gas cell sizes attain homogeneity, but when gravity is significant, the distribution of gas cells at the top, centre and bottom will differ from each other [41]. During proofing, as the gas cells expand under CO_2_ gas released by the leavening agent, coalescence and liquid drainage/separation from the dough may occur. However, the stability and coalescence of gas cells in the crumb are both set during thermal treatment (baking, frying or steaming). As dehydration occurs during frying, phase conversion from liquid to solid occurs causing gluten protein to reaggregate. This reaggregation gives a rigid structure to the gas cells, thus terminating coalescence [42].

### 3.5. Relationship between Microstructural Properties against Soxhlet Fat Extraction 

In order to categorise the relationship between the microstructure and oil absorption, POsox data was plotted against POia and porosity. The data presented non-linear relationships and were thus fitted to a cubic polynomial model, as established in the regression equations (Table 4). The effectiveness of the image analysis technique for oil absorption measurement was assessed by the R^2^ value of the regression equations. As established in Table 1, fried batter products were more porous, hence they retained more oil than fried dough.

#### 3.5.1. Oat Bran Fried Batter 

The strongest relationship was found in POsox vs. POia, where R^2^ = 0.9051 (Table 4), also corroborating the strong positive Pearson’s correlation found among both parameters (*p* < 0.001). Porosity vs. POsox (R^2^ = 0.7298) follows and could be as a result of differences in conversion parameters at macroscopic and microscopic levels of analysis, while porosity vs. POia (R^2^ = 0.7252) showed the weakest relationship, which could be attributed to product type and variation in the oil penetration of fried batter samples. 

#### 3.5.2. Oat Bran Fried Dough 

The relationship between POia, POsox and porosity of OB fried dough is shown in Table 4. The data was fitted to a third order (cubic) polynomial regression. The strongest relationship was found in porosity vs. POia (R^2^ = 0.8432), attributable to the similarity of the scale used in image analysis, hence the higher fitness of the data. Porosity vs. POsox was R^2^ = 0.7243 and the weakest was POia vs. POsox (R^2^ = 0.7011). Moreno and Bouchon [4] reported R^2^ = 0.8372 for a linear relationship between POia and POsox in a gluten-based fried matrix. 

#### 3.5.3. Wheat Bran Fried Dough 

Although both methods ranked the oil content of WB fried dough in similar ranges, the relationship between POia and POsox in WB fried dough products were the weakest (R^2^ = 0.7283). This weakness may be accounted for by the difference in the micron range scale in image analysis compared to the macro scale factors in Soxhlet extraction. This contradicts the report of Moreno and Bouchon [4], where POia and POsox ranked gluten-based fried products in the same manner, with a strong linear relationship. Porosity and POsox had the strongest regression coefficient (R^2^ = 0.9885), whereas porosity vs. POia was R^2^ =0.7555 (Table 4)

#### 3.5.4. Wheat Bran Fried Batter

In WB fried batter, the strongest to the weakest relationships were POia and porosity (R^2^ = 0.9521), POia and POsox (R^2^ = 0.8420) and POsox and porosity (R^2^ = 0.7077), as shown in Table 4. The Pearson’s correlation showed that an increase in porosity led to an increase in oil penetration (*p* < 0.01). It is worth noting that the relationships between fried batter ranked higher than fried dough and this may be because the POia of fried batter ranked higher than the Soxhlet data for the samples.

### 3.6. Crust Surface Roughness of Magwinya

**Figure 7:**
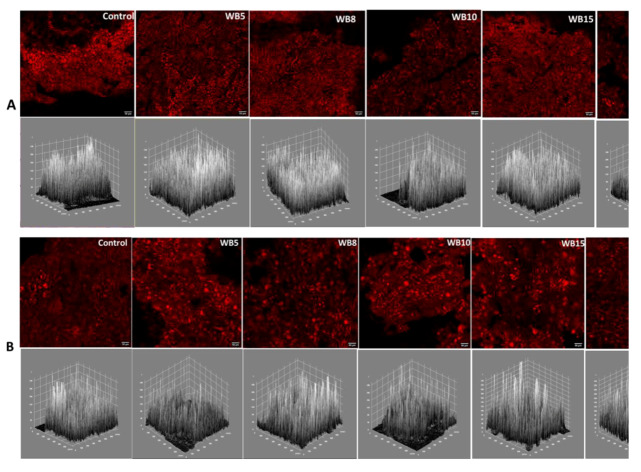
Crust confocal micrographs (top) of wheat bran fried batter (**A**) and fried dough (**B**) and their respective grey level intensity plots (bottom). Wheat bran (WB); values between five and 20 represent the concentration of bran in the sample formulation.

**Figure 8:**
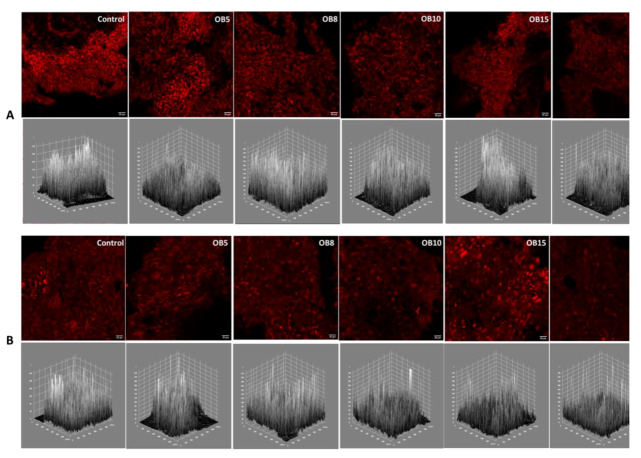
Crust confocal micrographs (top) of oat bran fried batter (**A**) and fried dough (**B**) and their respective grey level intensity plots (bottom). Oat bran (OB); values between five and 20 represent the concentration of bran in the sample formulation.

Crust micrographs and the grey level intensity maps of the crust micrographs of WB fried batter and fried dough is presented in Figure 7, while that of OB fried batter and dough is shown in Figure 8. The crust micrographs of fried products showed slight variations in terms of intensity. Fried dough had a lower intensity than fried batter. Structurally, the geometric appearance of fried batter showed closely knit cells, while the cells of fried dough were slightly larger, accounting for the lower intensity in the former. Grey level intensity maps of a product can reveal the nature of its surface. The surface plot maps were all jagged and closely related and had similar ranges of pixel intensity variation, except for a few differences seen in WBB8 and WBD8. The similarities may also be attributed to the fact that the close range of FD values in this study were within a narrow range which could be indicative of similarity in product formulation with minimal differences. In a comparison of the surface roughness of pumpkin and chocolate, Quevedo et al. [43] observed that pumpkin shell had a more jagged surface intensity than chocolate. Rahimi and Ngadi [13] also reported strong similarities in the intensity of the surface plots of fried batters made from wheat and rice flour. The use of fractal analysis has been applied to in quantifying the surface roughness of fried potato, chocolate, pumpkin [40], fried batter [13,14] and bread [29].

### 3.7. Surface Roughness Using Fractal Metrics

Fractal dimension is ranked on a value scale from one to three depending on the extent of deviation from regularity or as occupied in a Euclidean space [11]. The fractal dimension (FD) of fried dough and fried batter ranged from 2.56 to 2.76 and 2.55 to 2.67 across WB and OB samples, respectively (Table 5). These values were in a similar range to the report of Rahimi and Ngadi [13]. In WB samples, the FD of fried batter was higher than that of fried dough, although there was no statistical significance, indicating the former had a higher surface roughness than the latter. It appears WB had a similar effect on surface roughness of the two types of products. In OB samples, fried dough had a higher FD (*p* < 0.05), indicative of a rougher surface compared to fried batter. Thanatuksorn et al. [14] reported an increase in the fractal dimension of a fried wheat dough model, with an increase in the initial moisture content of fried batter. Compared to their study, a reverse trend was observed in our control sample (with no bran), where fried dough had a rougher surface than fried batter. Moreover, Thanatuksorn et al. [14] only determined the surface edge contours of the samples, whereas the entire crust surface was taken into consideration in this study (and not the contours), hence a possible reason for the difference in results. 

Measurement of surface roughness is crucial because it displays the irregularities of the crust, which may negatively impact oil absorption and consumer acceptance. Ghaitaranpour et al. [5] reported a maximum FD value of 1.97 for deep-fried doughnuts. This value was lower than the results in this study because of the extra dimension added to FD values in this study. We hypothesized that the surface roughness of fried batter would supersede that of fried dough due to the higher moisture loss in fried batter. This hypothesis can, however, be rejected based on the results obtained and the sample type and shape—*magwinya* being a thick and round fried food meant that development of surface texture varied at different rates due to the nature of its shape. The effect WB and OB had on surface roughness differed based on the initial moisture content, chemical composition of the fibres and difference in solubility of the bran, which imparted surface texture during frying. In addition, the higher surface roughness of fried dough could be attributed to the drier surface as a result of a lower initial moisture content. 

Multivariate analysis showed that the linear effect of bran concentration had a significant effect (*p* < 0.001) on FD, while bran type had no significant effect (*p* = 0.104). The main effect of initial moisture content and bran type were significant (*p* < 0.05) in relation to the fractal dimension values of the products. The interaction effect of bran type and initial moisture content (*p* < 0.001), bran type and bran concentration (*p* = 0.03) significantly affected FD. The interaction effect of the three independent variables (bran type, bran concentration and initial moisture content) all had no significant effect (*p* = 0.243) on FD. 

### 3.8. Correlation of FD to Surface Oil and Texture of Fried Products

Scatterplots revealed that the relationships between FD and crust properties (surface oil and hardness) are non-linear; regression models using a curve estimation function were adopted to observe and quantify the model. Some studies have shown that increased surface roughness had an impact on oil uptake through a positive linear correlation between the surface oil and fractal dimension [13,14]. In this study, the data between FD and crust properties were fitted to a polynomial cubic model, as shown by the regression equations and coefficient of determination (R^2^) in Table 5.

#### 3.8.1. Fractal Dimension vs. Surface Oil

Surface oil was plotted against FD values for each product type and the cubic polynomial model with the R^2^ values in the following order in Table 6: fried dough (WB: 0.98, OB: 0.97) and fried batter (WB: 0.96, OB: 0.88). Fried dough samples ranked higher in terms of the fitness of the data. The surface oil of fried dough was higher than in fried batter and this could be responsible for the higher R^2^ values of the former. 

#### 3.8.2. Fractal Dimension vs. Crust Hardness

Fractal dimension values were also plotted against crust hardness and the relationship also fitted a cubic polynomial model with the following R^2^ values: fried dough (WB: 0.56, OBM: 0.81) and fried batter (WB: 0.85, and OB: 0.99), as shown in Table 6. A significant correlation was only noticed in fried batter samples compared to fried dough. Fried batter ranked higher than fried dough in terms of surface roughness and crust hardness. Factors like frying time, temperature, product composition and initial moisture content, affect crust formation, which directly impacts the hardness of the food [44]. Fried batter was softer than fried dough because of the higher initial moisture content. 

## 4. Conclusions

This paper presents the first study on the quantitative analysis of *magwinya* crumb and crust properties determined from confocal micrographs. The use of distinct fluorescent dyes in sample preparation proved adequate for characterisation of oil penetration and structural changes in the samples using an image analysis technique, which revealed important knowledge about the relationship between oil uptake and the microstructure of *magwinya*. The results obtained from the image analysis were correlated to results from conventional Soxhlet extraction and the former could be used as an alternative analysis choice for future studies.

Cross-section micrographs of *magwinya* revealed notable differences in terms of oil distribution, depth of oil penetration, structure and pore properties of the fried products, thus emphasising the impact of ingredient formulation (water and bran variation) on oil penetration. The incorporation of oat and wheat bran in *magwinya* formulation reduced porosity and oil penetration. Fried batter was more porous than fried dough, owing to the higher initial moisture content of the former, which led to the increased evaporation of water, causing disproportionately heterogenous pore sizes (consisting mainly of larger ones). In comparison to the control sample, a reduction in porosity was observed for all samples. A cubic polynomial relationship was established between POia, porosity and POsox for magwinya crumb, as well as between FD and crust hardness and surface oil for the crust. The penetration of oil into the crumb was reduced and varied among the samples, with a reduction in initial moisture content and an increase in bran concentration (*p* < 0.05), although a minimal effect was observed for bran type. The utilisation of the double fluorescent staining protocol and multichannel CSLM observation used in this study was satisfactory for the observation and quantitative analysis of magwinya crumb and crust microstructure. Considering the information provided in this paper, there exist rich prospects for future studies and improvements to the work done.

## Figures and Tables

**Table 1:** Porosity and penetrated oil (PO) content determined by image analysis (POia) and PO by Soxhlet.

Bran Concentration (g)	Fried Batter (%)			Fried Dough (%)		
	POia	POSox	Porosity	POia	POSox	Porosity
**Control**	19.66 ^b^ ± 0.92	8.67 ^b^ ± 0.08	81.84 ^b^ ± 1.24	8.29 ^b^ ± 0.74	8.20 ^e^ ± 0.05	80.03 ^d^ ± 4.31
**OB5**	16.55 ^ab^ ± 0.47	9.47 ^c^ ± 0.08	67.10 ^a^ ± 2.62	5.56 ^a^ ± 0.34	6.67 ^bc^ ± 0.16	69.21 ^c^ ± 1.35
**OB8**	16.03 ^ab^ ± 1.21	8.80 ^b^ ± 0.14	73.66 ^ab^ ± 0.84	14.28 ^d^ ± 0.37	7.13 ^cd^ ± 0.11	54.94 ^a^ ± 1.53
**OB10**	14.52 ^a^ ± 0.61	7.47 ^a^ ± 0.08	76.77 ^b^ ± 1.79	11.31 ^c^ ± 0.20	6.33 ^b^ ± 0.15	58.58 ^ab^ ± 1.41
**OB15**	18.22b ^ab^ ± 0.60	8.60 ^b^ ± 0.30	80.68 ^b^ ± 1.05	18.92 ^e^ ± 0.48	5.27 ^a^ ± 0.06	65.93 ^bc^ ± 2.79
**OB20**	14.97 ^a^ ± 1.63	7.87 ^a^ ± 0.11	65.91 ^a^ ± 1.68	11.73 ^c^ ± 0.42	7.53 ^d^ ± 0.08	60.76 ^ab^ ± 2.68
**Control**	19.66 ^c^ ± 0.74	8.53 ^b^ ± 0.46	81.84 ^c^ ± 1.24	8.29 ^bc^ ± 0.16	7.07 ^c^ ± 0.31	80.03 ^d^ ± 4.31
**WB5**	13.18 ^a^ ± 0.47	9.93 ^c^ ± 0.61	55.71 ^ab^ ± 3.92	4.57 ^a^ ± 0.22	8.00 ^d^ ± 0.26	52.15 ^a^ ± 0.59
**WB8**	13.81 ^ab^ ± 0.30	7.67 ^a^ ± 0.08	53.12 ^a^ ± 2.69	9.29 ^c^ ± 0.03	7.73 ^d^ ± 0.57	73.08 ^cd^ ±1.22
**WB10**	16.00 ^b^ ± 0.53	9.87 ^c^ ± 0.09	70.56 ^bc^ ± 2.10	6.43 ^b^ ± 0.03	6.07 ^b^ ± 0.31	54.68 ^ab^ ± 2.69
**WB15**	13.52 ^a^ ± 0.31	10.13 ^c^ ± 0.31	75.69 ^c^ ±2.57	7.58 ^bc^ ± 0.11	6.20 ^b^ ± 0.40	56.88 ^ab^ ± 2.33
**WB20**	15.31 ^b^ ± 0.30	8.73 ^b^ ± 0.98	76.05 ^c^ ± 2.23	7.94 ^bc^ ± 0.17	5.00 ^a^ ± 0.15	65.15 ^bc^ ± 2.66

Results are shown as mean ± standard deviation (*n* = 4). Values in the same column with different superscript for each bran type are significantly different from each other (*p* ≤ 0.05) using Tukey’s honestly significant difference test. Oat bran (OB) and wheat bran (WB). Values 5–20 represent the bran concentration in the product formulation.

**Table 2:** Regression analysis showing main and interaction effects of independent variables on percentage porosity and penetrated oil by image analysis.

Source	Dependent Variable (%)	Type III Sum of Squares	df	Mean Square	F	Significance ^c^
Corrected Model	PorosityPOia	29879 ^a^	23	1299.09	20.04	<0.001
		4661 ^b^	23	202.67	55.83	<0.001
Intercept	Porosity	825,377	1	825,377	12,732.53	<0.001
	POia	27,124	1	27,124.72	7471.72	<0.001
X	Porosity	88	1	88.427	1.36	0.244
	POia	1000	1	1000.281	275.54	<0.001
Y	Porosity	6938	1	6938.65	107.04	<0.001
	POia	1104	1	1104.91	304.36	<0.001
Z	Porosity	13,384	5	2676.86	41.29	<0.001
	POia	715	5	143.034	39.4	<0.001
X * Y	Porosity	2256	1	2256.54	34.81	<0.001
	POia	1.56	1	1.56	0.43	0.513
X * Z	Porosity	1005	5	201.12	3.10	0.01
	POia	533	5	106.52	29.34	<0.001
Y * Z	Porosity	2927	5	585.48	9.03	<0.001
	POia	1047	5	209.32	57.66	<0.001
X * Y * Z	Porosity	3278	5	655.62	10.11	<0.001
	POia	261	5	52.05	14.34	<0.001
Error	Porosity	10,891	168	64.82		
	POia	610	168	3.63		
Total	Porosity	866,147	192			
	POia	32,396	192			
Corrected Total	Porosity	40,769	191			
	POia	5271	191			

df = degree of freedom, X = bran type, Y = initial moisture content (mL), Z = bran concentration (g). Interaction effect of variables is denoted by X * Y, X * Z, Y * Z and X * Y * Z. Penetrated oil by image analysis (POia) (%). (a) R^2^ = 0.733 (b) R^2^ = 0.884 (c) computed using alpha = 0.05.

**Table 3:** Pore properties of magwinya samples, as influenced by the addition of water and bran variation.

Bran Concentration (g)	Fried Dough					
	Particle Count (ΣP)	Total Area (×10^4^ µm^2^)	Average Size (µm^2^)	Perimeter (µm)	Circularity (C)	Solidity (S)
Control	18	12	728	150	0.29	0.73
OB5	44	27	6120	948	0.10	0.64
OB8	44	23	5216	1119	0.15	0.67
OB10	45	12	2746	696	0.13	0.65
OB15	60	15	2503	435	0.18	0.73
OB20	67	22	3303	551	0.15	0.69
WB5	76	23	3085	611	0.14	0.64
WB8	48	29	6036	566	0.19	0.77
WB10	59	28	4756	545	0.16	0.70
WB15	49	26	5237	389	0.24	0.75
WB20	59	22	3703	606	0.14	0.66
**Fried batter**						
Control	28	25	3470	343	0.42	0.81
OB5	20	42	2102	861	0.15	0.72
OB8	40	17	4207	415	0.34	0.84
OB10	34	20	5846	508	0.19	0.77
OB15	62	17	2692	430	0.16	0.67
OB20	62	23	3690	495	0.18	0.72
WB5	59	17	2836	319	0.16	0.66
WB8	41	43	10,288	564	0.20	0.74
WB10	24	38	15,662	544	0.19	0.73
WB15	69	27	3890	558	0.14	0.66
WB20	73	25	3361	311	0.22	0.72

Oat and wheat bran (OB and WB, respectively); values between five and 20 represent the concentration of bran in the sample formulation.

**Table 4:** Cubic polynomial regression equation for plots of image properties and Soxhlet extraction.

Fried Dough	Porosity vs. POia	POia vs. POSoxhlet	POSoxhlet vs. Porosity
OB	y = 0.0976x^3^ − 3.3741x^2^ + 34.041x − 30.943	y = −1.4146x^3^ + 32.453x^2^ − 242.33x + 601.78	y = 5.757x^3^ − 110.56x^2^ + 698.29x − 1386.4
R^2^	0.84	0.70	0.72
WB	y = −1.1344x^3^ + 24.837x^2^ − 169.76x + 417.88	y = 0.0276x^3^ − 0.2017x^2^ − 1.3834x + 15.904	y = −16.047x^3^ + 309.49x^2^ − 1957.3x + 4120.2
R^2^	0.76	0.73	0.99
Fried batter			
OB	y = −0.6708x^3^ + 35.179x^2^ − 608.8x + 3551	y = 0.0603x^3^ − 3.2654x^2^ + 58.596x − 339.77	y = −22.873x^3^ + 577.36x^2^ − 4839.6x + 13544
R^2^	0.73	0.91	0.71
WB	y = 0.0001x^3^ + 0.0038x^2^ − 2.3198x + 105.18	y = 6.1133x^3^ − 164.02x^2^ + 1457.5x − 4274.8	y = 0.0009x^3^ − 0.2033x^2^ + 14.426x − 325.69
R^2^	0.95	0.84	0.71

Penetrated oil by image analysis and Soxhlet extraction (POia and POSoxhlet, respectively). Oat and wheat bran (OB and WB, respectively).

**Table 5:** Fractal dimension (FD) values of fried products.

Bran Concentration (g)	Fried Dough	Fried Batter	Significance
Control	2.69 ^b^ ± 0.13	2.58 ^ab^ ± 0.04	0.02
OB5	2.73 ^b^ ± 0.05	2.55 ^a^ ± 0.04	0.001
OB8	2.74 ^b^ ± 0.02	2.62 ^bc^ ± 0.05	0.01
OB10	2.76 ^b^ ± 0.08	2.61 ^abc^ ± 0.05	0.01
OB15	2.57 ^a^ ± 0.08	2.58 ^ab^ ± 0.03	0.95
OB20	2.73 ^b^ ± 0.04	2.66 ^c^ ± 0.04	0.05
Control	2.69 ^c^ ± 0.13	2.58 ^a^ ± 0.04	0.02
WB5	2.67 ^bc^ ± 0.03	2.64 ^a^ ± 0.09	0.53
WB8	2.57 ^ab^ ± 0.09	2.64 ^a^ ± 0.06	0.30
WB10	2.66 ^abc^ ± 0.02	2.65 ^a^ ± 0.07	0.95
WB15	2.56 ^a^ ± 0.07	2.67 ^a^ ± 0.09	0.12
WB20	2.63 ^abc^ ± 0.06	2.64 ^a^ ± 0.05	0.60

Results are shown as mean ± standard deviation (*n* = 4). Values with same superscripts down the column for each bran type show significance (*p* < 0.05) using Tukey’s HSD test. Significance values in the third column show significant difference between fried batter and dough using independent samples *T*-test at *p* < 0.05. Oat and wheat bran (OB and WB, respectively); values between five and 20 represent the concentration of bran in the sample formulation. Fractal dimension is dimensionless.

**Table 6:** Cubic polynomial regression equation for plots of fractal dimension vs. surface oil and crust hardness.

Sample	Cubic Polynomial Regression Model Equations	R^2^
WB fried dough	A = 67.39B^3^ − 51.454B^2^ + 12.752B + 1.6384	0.9822
	Y = −3E + 06X^3^ + 2E+07X^2^ − 5E+07X + 4E+07	0.5615
WB fried batter	A = 653.52B^3^ − 335.94B^2^ + 54.869B − 0.2328	0.9568
	Y = −3E−10X^3^ + 1E−06X^2^ − 0.0008X + 2.5568	0.8451
OB fried dough	A = 7.3707B^3^ − 14.254B^2^ + 5.9505B + 2.0324	0.9736
	Y = 1E − 09X^3^ − 7E−06X^2^ + 0.0155X − 8.7206	0.8072
OB fried batter	A = −291.74B^3^ + 158.88B^2^ − 28.375B + 4.2429	0.8771
	Y = 465122X^3^ − 4E + 06X^2^ + 9E+06X − 8E + 06	0.9853

Where A and Y = fractal dimension, B = surface oil, X = crust hardness. Oat and wheat bran (OB and WB, respectively).
